# The Ciliopathy Gene *ahi1* Is Required for Zebrafish Cone Photoreceptor Outer Segment Morphogenesis and Survival

**DOI:** 10.1167/iovs.16-20326

**Published:** 2017-01

**Authors:** Emma M. Lessieur, Joseph Fogerty, Robert J. Gaivin, Ping Song, Brian D. Perkins

**Affiliations:** 1 Department of Ophthalmic Research, Cole Eye Institute, Cleveland Clinic, Cleveland, Ohio, United States; 2 Department of Molecular Medicine, Cleveland Clinic Lerner College of Medicine of Case Western Reserve University, Cleveland, Ohio, United States

**Keywords:** photoreceptor, cilia, Joubert syndrome, zebrafish, disc morphogenesis

## Abstract

**Purpose:**

Joubert syndrome (JBTS) is an autosomal recessive ciliopathy with considerable phenotypic variability. In addition to central nervous system abnormalities, a subset of JBTS patients exhibit retinal dystrophy and/or kidney disease. Mutations in the *AHI1* gene are causative for approximately 10% of all JBTS cases. The purpose of this study was to generate *ahi1* mutant alleles in zebrafish and to characterize the retinal phenotypes.

**Methods:**

Zebrafish *ahi1* mutants were generated using transcription activator-like effector nucleases (TALENs). Expression analysis was performed by whole-mount in situ hybridization. Anatomic and molecular characterization of photoreceptors was investigated by histology, electron microscopy, and immunohistochemistry. The optokinetic response (OKR) behavior assay was used to assess visual function. Kidney cilia were evaluated by whole-mount immunostaining.

**Results:**

The *ahi1^lri46^* mutation in zebrafish resulted in shorter cone outer segments but did not affect visual behavior at 5 days after fertilization (dpf). No defects in rod morphology or rhodopsin localization were observed at 5 dpf. By 5 months of age, cone degeneration and rhodopsin mislocalization in rod photoreceptors was observed. The connecting cilium formed normally and Cc2d2a and Cep290 localized properly. Distal pronephric duct cilia were absent in mutant fish; however, only 9% of *ahi1* mutants had kidney cysts by 5 dpf, suggesting that the pronephros remained largely functional.

**Conclusions:**

The results indicate that Ahi1 is required for photoreceptor disc morphogenesis and outer segment maintenance in zebrafish.

Cilia are microtubule-based organelles that emerge from the apical membrane of virtually all eukaryotic cells.^1^ Motile cilia facilitate fluid movement, while nonmotile (primary) cilia function as sensory organelles that allow the cell to perceive and respond to changes in the extracellular environment. The outer segment of vertebrate photoreceptors is a modified primary cilium that has been specialized for photon detection.^2^ Hundreds of tightly packed disc membranes, which contain the protein components necessary for phototransduction, fill the photoreceptor outer segment. The disc membranes stack horizontally within the outer segment and align perpendicular to the microtubule axoneme. The outer segment attaches to the photoreceptor inner segment via the connecting cilium, which is similar to the transition zone of other cilia,^2^ and proteins destined for the outer segment must traverse the connecting cilium to become incorporated into the disc membranes. Defects in trafficking machinery or ciliary structure often will result in abnormal disc assembly, protein mislocalization, outer segment degeneration, and photoreceptor death manifesting in retinal dystrophy.^3–5^

Defects in cilia biogenesis and function also have been implicated in a variety of human developmental disorders commonly known as ciliopathies,^6^ which include primary ciliary dyskinesia, Bardet-Biedl syndrome (BBS), Senior-Løken syndrome (SLS), Meckel-Grueber syndrome, and Joubert syndrome (JBTS). The clinical manifestations of ciliopathies result from dysfunction in motile as well as nonmotile cilia and, therefore, impact a myriad of organs, including the retina, central nervous system, kidney, liver, and pancreas.^6,7^

Joubert syndrome is a rare autosomal recessive ciliopathy with considerable genotypic and phenotypic variability.^8^ Mutations in at least 27 genes have been linked to JBTS and all of these genes encode proteins localizing to the cilium.^8^ The defining characteristic of JBTS is the “molar tooth sign” observed by MRI, which results from cerebellar vermis hypoplasia.^9,10^ Affected individuals also display delayed motor development, abnormal breathing patterns, cognitive difficulties, and oculomotor apraxia. In addition to impairments of the central nervous system, retinal dystrophy is present in approximately 30% of patients and kidney disease is present in approximately 25% of JBTS cases.^8,11^ Analysis of over 330 JBTS cases found a moderate association for the co-occurrence of retinal dystrophy and kidney disease (odds ratio 3.0).^8^ Other clinical manifestations include liver fibrosis, coloboma, polydactyly, and skeletal dystrophy.

Mutations in the *Abelson helper integration site 1 (AHI1)* gene cause approximately 7% of known cases of JBTS,^12–14^ but predicting genotype–phenotype correlations remains difficult. Retinal dystrophy is more strongly correlated with mutations in *AHI1* than that of most other JBTS genes, but retinal involvement remains variable even with lesions in *AHI1*.^8^ In addition to being a causative gene for JBTS, *AHI1* alleles also can function as modifiers of retinal degeneration in nephronophthisis, a ciliopathy characterized by kidney dysplasia.^15,16^ *AHI1* encodes a multidomain protein also known as jouberin, which consists of an N-terminal coiled-coil domain, seven WD40 repeats, and a C-terminal SH3 domain. Ahi1 localizes to the basal body of primary cilia and Ahi1 function is required for ciliogenesis in cultured cells.^17^ Deletion of *Ahi1* in mice resulted in high mortality at birth. In surviving animals, photoreceptors failed to generate outer segments, but axonemes and rudimentary connecting cilia were present before photoreceptor degeneration.^15,18^ Although photoreceptor death and perinatal lethality in *Ahi1*-null mice was consistent with deficits in cilia function, the severity of these phenotypes limits their use for studying genetic interactions that modulate the degree of retinal degeneration. Furthermore, a recent study reported that four individuals harbored homozygous truncating mutations that eliminated the SH3 domain, and yet they did not exhibit any signs of JBTS.^19^ To better understand how *AHI1* variants affect development and retinal function, genetically tractable in vivo animal models are needed.

We report the generation and characterization of *ahi1* mutant zebrafish. Zebrafish *ahi1* mutants exhibit phenotypes consistent with the oculorenal disease in JBTS. Loss of Ahi1 resulted in disrupted cone photoreceptor outer segments and distal pronephric cilia at larval stages. Although photoreceptor axonemal architecture was unaltered, outer segment disc membranes were severely disorganized and considerable vesiculation was observed near the connecting cilium. Cone degeneration and rhodopsin mislocalization was observed in a small group of 3- to 5-month-old animals that escaped lethality. Our results suggested that Ahi1 is required for photoreceptor outer segment morphogenesis and maintenance.

## Materials and Methods

### Zebrafish Husbandry

Adult zebrafish were maintained and raised on an Aquatic Habitats recirculating water system (Pentair; Apopka, FL, USA) in a 14:10-hour light-dark cycle. The Cleveland Clinic Institutional Animal Care and Use Committee (IACUC) approved all experimental procedures and all studies conformed to the ARVO Statement for the Use of Animals in Ophthalmic and Vision Research. For basal body localization experiments, we used the transgenic line *Tg(5actb2:cetn2-GFP)^cu6^*, abbreviated *Tg(centrin:GFP)*, which expresses a centrin2-GFP fusion protein from the β-actin promoter.^20^

### Design of Transcription Activator-Like Effector Nucleases (TALENs) Targeting *ahi1* in Zebrafish

Transcription activator-like effector nucleases targeting zebrafish *ahi1* were designed with TALENT software (available in the public domain at https://talent.cac.cornell.edu/TALENT/). The Golden Gate assembly method^21^ was used to generate the TALEN constructs. We synthesized 5′-capped mRNA encoding the TALENs using the Sp6 mMESSAGE mMACHINE Kit (Ambion; Thermo Fisher Scientific, Waltham, MA, USA) and microinjected 100 pg of mRNA into wild-type zebrafish embryos at the one cell stage.

### Genotyping

The *ahi1^lri46^* mutants were genotyped by PCR using primers targeting *ahi1* exon 5 (5′-TGCAGTCAGGCTGAAGTGTC-3′, 5′-TTTCTTCTTCACTTTGGGTTTGA-3′) to generate a 102 base pair (bp) product (wild-type) or a 95 bp product (mutant). PCR products were resolved on a 3% agarose gel.

### In Situ Hybridization

In situ hybridization was performed on fixed tissue from 1-cell stage to 5 days after fertilization (dpf) larvae as previously reported.^22^ A zebrafish *ahi1* cDNA clone was purchased from GE Dharmacon (clone ID 9038651; Lafayette, CO, USA), and antisense probes were generated with the mMESSAGE mMACHINE T7 Kit (Ambion; Thermo Fisher Scientific).

### Light and Electron Microscopy

Larvae were bisected through the swim bladder. Tails were used for genotyping while heads were prepared for transmission electron microscopy. Enucleation of adult eyes was performed after euthanasia in fish water at 4°C. Briefly, all tissues were fixed for 1 hour at room temperature in 0.08 M cacodylate buffer containing 2% paraformaldehyde and 2% glutaraldehyde. Samples then were washed with cacodylate buffer and postfixed in 1% osmium tetroxide for 1 hour at 4°C. Samples were washed again and then dehydrated in a graded methanol series before embedding them in Embed-812/DER736 (Electron Microscopy Sciences; Hatfield, PA, USA), using acetonitrile as a transition solvent. Tails were used for genomic DNA extraction and genotyping as described above. Adult animals were genotyped by fin clips at 2 months old. Semithin sections were made with a Leica EM UC7 ultramicrotome (Leica Microsystems GmbH, Vienna, Austria), stained with toluidine blue, and imaged with a Zeiss AxioImager.Z2 (Carl Zeiss Microscopy, Thornwood, NY, USA). Ultrathin sections were stained with uranyl acetate and lead citrate following standard procedures, and electron microscopy was performed on a Tecnai G2 Spirit BioTWIN 20-120 kV digital electron microscope (FEI Company, Hillsboro, OR, USA). Micrographs were acquired with a Gatan image filter and an Orius 832 CCD Camera (Gatan, Inc., Pleasanton, CA, USA).

### Optokinetic Response (OKR)

Optokinetic response measurements were conducted between 12 and 6 PM using the VisioTracker system (VisioTracker 302060 Series; TSE Systems GmbH, Bad Homburg, Germany). Contrast sensitivity was assessed as described previously.^23^ For the spatial frequency response function,^24^ the contrast was held constant at 70% and we tested stimuli of 0.02, 0.04, 0.06, 0.08, 0.12, and 0.16 cycles/deg by first increasing and then decreasing the frequency. Each spatial frequency stimulus was presented for 3 seconds before reversing direction for another 3 seconds to minimize saccade frequency. All OKR stimuli were presented with a constant angular velocity of 7.5° per second. The genotypes of individual larvae were confirmed following OKR tests.

### Immunohistochemistry and Fluorescence Imaging

Samples were fixed at the designated time points. Fixation protocols varied depending on the primary antibodies being used. For zpr1, zpr3, 1D1, peanut agglutinin (PNA), Ift88 and acetylated tubulin, samples were fixed in 4% paraformaldehyde in ×0.8 PBS at 4°C overnight. For Cep290 staining, heads were fixed in 4% paraformaldehyde in ×0.8 PBS at 4°C for a maximum of 2 hours. For Cc2d2a staining, heads were fixed in 2% trichloroacetic acid (TCA) in double distilled water for 2 hours at room temperature. All samples were cryoprotected in 30% sucrose overnight. Cryosections (10 μM) were cut and dried at room temperature overnight. Blocking solution (1% BSA, 5% normal goat serum, 0.2% Triton-X-100, 0.1% Tween-20 in 1× PBS) was applied for 2 hours in a dark, humidified chamber. Primary antibodies were diluted in blocking solution as follows: zpr1 and zpr3 (1:200; Zebrafish International Resource Center, University of Oregon, Eugene, OR, USA), 1D1^25^ (1:100; gift from James Fadool), acetylated-α-tubulin (1:5000; Sigma 6-11-B1), Ift88 (1:7500),^26^ Cep290 (1:100; gift from Iain Drummond, Massachusetts General Hospital, Boston, MA, USA), and Cc2d2a^27^ (1:20; gift from Ruxandra Bachmann-Gagescu, University of Zurich, Zurich, Switzerland). Conjugated secondary antibodies were purchased from Invitrogen Life Technologies (Carlsbad, CA, USA) and used at 1:500 dilutions and 4′,6-diamidino-2-phenylendole (DAPI; 1:1000) was used to label nuclei. For whole mount analysis of the kidney cilia, 36 hours after fertilization (hpf) embryos were fixed in Dent's fixative (80% methanol, 20% dimethyl sulfoxide [DMSO]) overnight at room temperature. The next day, whole mount immunostaining experiments were performed as follows: embryos were rehydrated in a graded series of MeOH:PBST washes for 15 minutes each at room temperature, and incubated in blocking solution (PBS, 1% DMSO, 0.5% Tween 20, 1% BSA, 10% normal goat serum) overnight at 4°C. Embryos were incubated with a mouse anti-acetylated-α-tubulin primary antibody (1:500) diluted in incubation solution (PBS, 1% DMSO, 0.5% Tween 20, 2% normal goat serum) for a minimum of 36 hours at 4°C. Embryos then were washed 4 times with incubation solution for 30 minutes each and incubated with a fluorophore-conjugated secondary antibody (1:1000) diluted in incubation solution overnight at 4°C. Following antibody staining, embryos were washed again with incubation solution, heads were removed for genotyping, and tails were mounted on slides for imaging. Optical sections were obtained with a Zeiss Axio Imager.Z2 fluorescent microscope fitted with the Apotome.2 for structured illumination (Carl Zeiss Microscopy). ImageJ was used on PNA-stained sections to measure cone outer segment length and on 1D1-stained sections to measure rod outer segment area, as well as to create image panels.

### Rescue by mRNA Injection

A cDNA clone encoding full-length, wild-type human *Ahi1* was purchased from OriGene Technologies (clone SC114030; Rockville, MD, USA). A codon switch encoding the *ahi1^R589X^* mutation was introduced by site-directed mutagenesis (GeneArt Site-Directed Mutagenesis System; Thermo Fisher Scientific). Capped mRNAs were synthesized using the mMessage mMachine Sp6 transcription kit (Thermo Fisher Scientific). Messenger RNA was injected into 1-cell stage embryos with 0.05% phenol red. Embryos were evaluated for phenotypic rescue by immunostaining kidney cilia in whole 36 hpf embryos with acetylated α tubulin antibodies. As kidney cilia are stereotypically longer than other cilia in the developing embryo, we counted all cilia in the distal kidney longer than 3 μm to ensure that the analysis was tissue-specific.

### Statistics

Graphs were generated using Prism6 (GraphPad Software, San Diego, CA, USA). Statistical analysis was performed using 2-tailed and unpaired Student's *t*-test. For the rescue experiment, a 1-way ANOVA with a Tukey's Multiple Comparison test was used. For all tests, *P* values less than 0.05 were considered significant.

## Results

### Zebrafish *ahi1* Mutants Exhibit a Ciliopathy Phenotype

**Figure 1:**
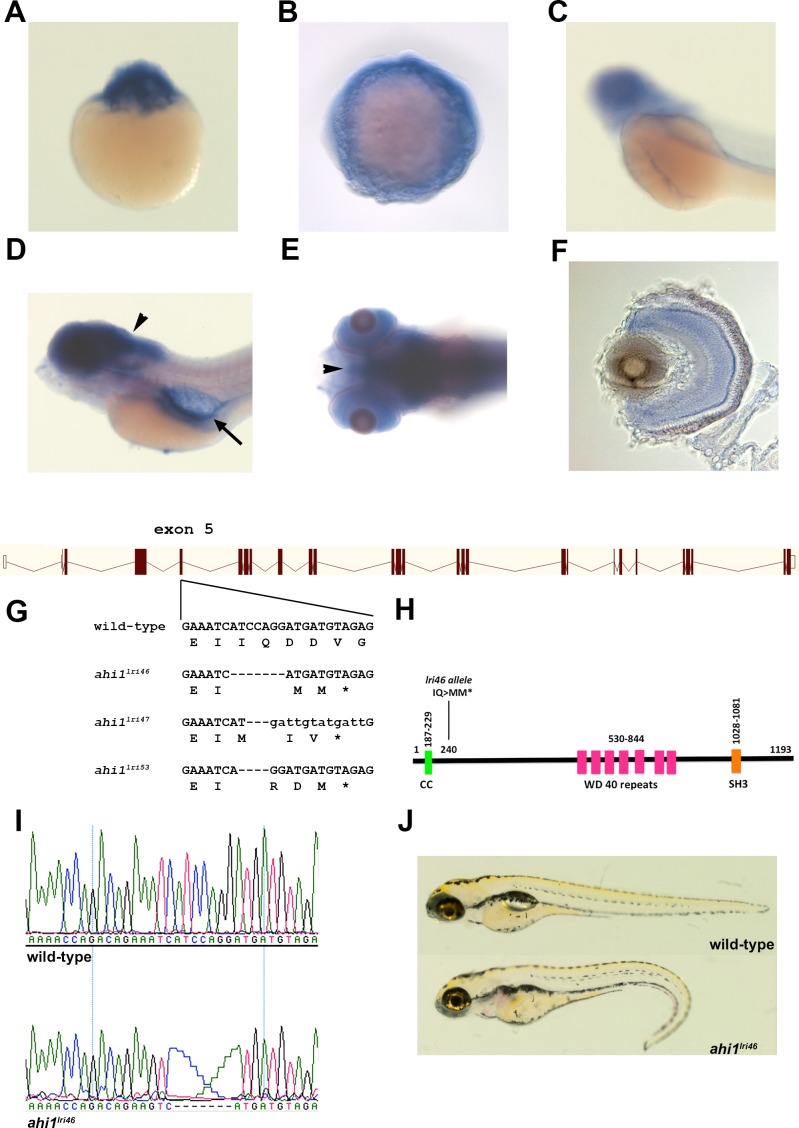
Generation of *ahi1* mutant zebrafish. (**A**–**C**) Whole-mount in situ hybridization from 1 cell (**A**), 10 hpf (**B**), and 48 hpf (**C**), showing expression of *ahi1*. (**D**, **E**) Lateral and ventral views showing 5 dpf zebrafish stained by whole-mount in situ hybridization. *ahi1* expression was observed throughout the central nervous system (*arrowheads*) and pronephros (*arrow*). (**F**) A transverse section shows *ahi1* expression throughout the retina at 5 dpf. (**G**) Illustration of *ahi1* genomic architecture. The wild-type sequence and TALENs induced *ahi1* mutant alleles are shown. Wild-type nucleotides are in *upper case*. Deleted nucleotides are denoted as *dashes* while inserted nucleotides are in *lower case letters*. (**H**) Schematic protein structure of Ahi1 illustrating the location of the coiled-coil (CC) domain, WD40 repeats, and SH3 domain. The *ahi1^lri46^* is a frame shift mutation and encodes a truncated protein lacking the 7 WD 40 repeats and the SH3 domain. (**I**) Chromatograms of Sanger sequencing reactions of *ahi1* wild type (wild-type) and homozygous mutant (*ahi1^lri46^*) zebrafish. (**J**) Lateral view of representative wild-type and *ahi1^lri46^* homozygous mutant at 5 dpf.

We first examined the spatial expression of *ahi1* at multiple time points during development using whole-mount in situ hybridization. We detected maternally deposited transcripts of *ahi1* in 1-cell embryos (Fig. 1A). Expression of *ahi1* was observed in 10 hpf embryos and in 48 hpf and 5 dpf larvae (Figs. 1B–E). As previously reported,^22^ we observed expression of *ahi1* throughout the central nervous system (CNS), eye and pronephros (Figs. 1D, 1E) at 5 dpf in zebrafish larvae. Within the eye, *ahi1* was most abundantly expressed in the inner retina (Fig. 1F). Photoreceptor expression was not readily observed, but levels of *ahi1* may be below the level of detection in 5 dpf larvae.

To investigate the function of *ahi1,* we generated mutant zebrafish using TALENs.^21^ Transcription activator-like effector nucleases were designed against a target in exon 5 of the primary splice variant listed in Ensembl (ENSDARE00000761551). This exon is conserved among all the predicted zebrafish splice variants. Truncating mutations in exon 5 should result in the loss of the WD40 repeats and SH3 domain of the protein. The mRNAs encoding the *ahi1* TALENs were injected into 1-cell embryos and adult founders were identified by screening offspring for mutations by high-resolution melt analysis.^28^ Three *ahi1* mutant alleles were identified and confirmed by sequencing (Fig. 1G). The *ahi1^lri46^* mutation consisted of a 7 bp deletion, which generated a stop codon at amino acid 240 (Figs. 1G–I). The *ahi1^lri47^* mutation was a 3 bp deletion with a 12 bp insertion, which resulted in 3 new amino acids and a termination codon at amino acid 241. The *ahi1^lri53^* mutation produced a 4 bp deletion that also resulted in a stop codon at amino acid 241. All three mutants resulted in identical photoreceptor phenotypes (data not shown), and the *ahi1^lri46^* mutant was used for all subsequent analyses.

The gross phenotype of *ahi1* mutants was similar to other zebrafish mutants with defects in cilia formation and function.^26,29^ At 5 dpf, all *ahi1^lri46^* mutants had a ventral tail curvature and lacked swim bladders (Fig. 1J). Pericardial edema and hydrocephaly was observed in <2% of the mutants. All mutants had normal otolith numbers and exhibited left-sided expression of the nodal-related gene *southpaw* (data not shown), suggesting that ciliogenesis was not abrogated in all tissues. Only 9% (3 of 32) of mutant animals developed kidney cysts by 4 dpf. The morphologic phenotype was consistently observed in 25% of the progeny from crosses of heterozygous parents as expected for Mendelian inheritance, with mutants surviving to at least 10 dpf. Approximately 97% of *ahi1^lri46^* mutants died before metamorphosis, while approximately 2.5% of *ahi^lri46^* mutants (5 of 186) escaped lethality and survived to at least 3 months of age, but not longer than 5 months of age. These animals exhibited mild scoliosis (Supplementary Fig. S1), a phenotype previously linked to defective cilia.^30^

### Distal Pronephric Cilia Are Absent in *ahi1^lri46^* Mutants

**Figure 2:**
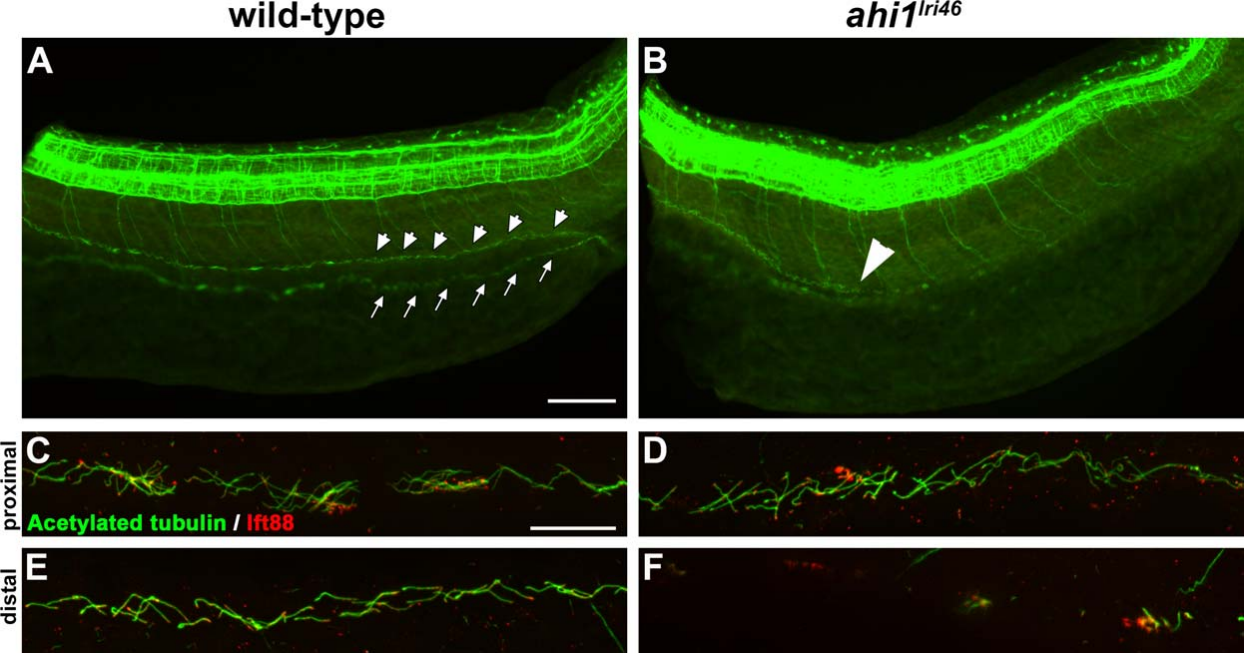
Loss of *ahi1* affects other ciliated tissues. (**A**, **B**) Anti-acetylated tubulin whole-mount immunostaining (*green*) of kidney duct in wild-type and *ahi1^lri46^* mutants at 36 hpf. Anterior is to the *left* in both images. Cilia line the pronephric duct (*small arrowheads*) in wild-type animals. The contralateral duct also can be observed (*small arrows*). Cilia are absent in the middle and distal pronephros in *ahi1^lri46^* mutants (*arrowhead* in [**B**] indicates the proximal boundary where cilia are missing). (**C**, **D**) In the proximal region, mutant cilia are not qualitatively different from wild-type controls. (**E**, **F**) Cilia in the distal region of the pronephros are absent in mutant animals when compared to controls. Ift88 immunostaining (*red*) was used to mark the ciliary transition zone. *Scale bars*: 50 μm (**A**, **B**) and 20 μm (**C**–**F**).

As kidney disease is a common feature of JBTS, and because we observed a minor but significant incidence of kidney cysts in mutant animals, we investigated whether renal cilia were affected during early development. To answer this question, we performed whole-mount immunostaining with antibodies for acetylated α-tubulin and Ift88 on 36 hpf embryos (Fig. 2). In wild-type animals, cilia line the entire pronephric duct, making the structure easily discernible in whole mount embryos (Fig. 2A, arrows, arrowheads). In all *ahi1^lri46^* mutants examined (*n* = 11), tubulin immunoreactivity was absent in the distal half of the duct (Fig. 2B). At higher magnification, cilia in the proximal duct of *ahi1^lri46^* mutants appeared similar to those observed in wild-type samples (Figs. 2C, 2D), but were completely absent in the distal half (Figs. 2E, 2F). Interestingly, the fact that kidney cysts are rarely observed in mutants, despite the universal lack of distal cilia, suggests that the pronephros is largely functional.

### Outer Segments of *ahi1^lri46^* Mutants Are Disorganized

**Figure 3:**
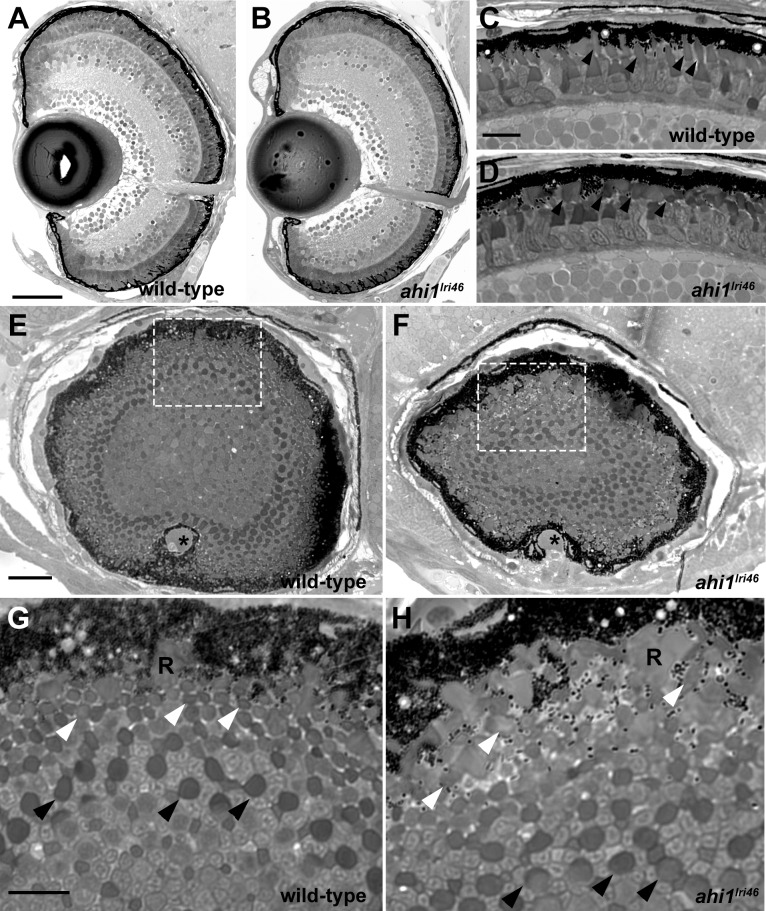
Histologic survey of retinas of 5 dpf wild-type and *ahi1^lri46^* larvae (**A**–**D**) Transverse sections of 5 dpf wild-type and *ahi1^lri46^* eyes. Photoreceptor outer segments (*arrowheads*) were shorter and less organized in *ahi1^lri46^* mutants. (**E**–**H**) Sagittal sections of wild-type and *ahi1^lri46^* eyes, taken at the posterior pole just dorsal to the optic nerve (*). Indicated regions (*white dashed boxes*) in (**E**, **F**) are shown at higher magnification in (**G**, **H**). *Black arrows* indicate the larger caliber, more proximal UV cone outer segments. *White arrows* indicate the smaller caliber, more distal *red*, *green*, and *blue* cone outer segments. R, rod outer segments. *Scale bars*: 50 μm (**A**, **B**), 10 μm (**C**, **D**, **G**, **H**) and 25 μm (**E**, **F**).

We next completed a histologic analysis to examine retinal morphology of 5 dpf larvae. In transverse semithin plastic sections, the *ahi1^lri46^* mutants exhibited normal retinal lamination (Figs. 3A, 3B) indicating that retinal development does not require *ahi1*. At higher magnification, the photoreceptor outer segments of *ahi1^lri46^* mutants were shorter and lacked the ordered, columnar organization observed in wild-type siblings (arrowheads in Figs. 3C, 3D). This phenomenon was more readily observed in sagittal sections through the extreme posterior pole of the eye, where cone outer segments were visible as a regular array of dark circles in wild-type samples (Figs. 3E, 3G, arrowheads). In *ahi^lri46^* mutant samples, the highly organized array of outer segments was lost, particularly that of the more distal cone types, and instead many of them appeared more ellipsoid, suggesting that they are misshapen or improperly oriented (Figs. 3F, 3H, white arrowheads).

**Figure 4:**
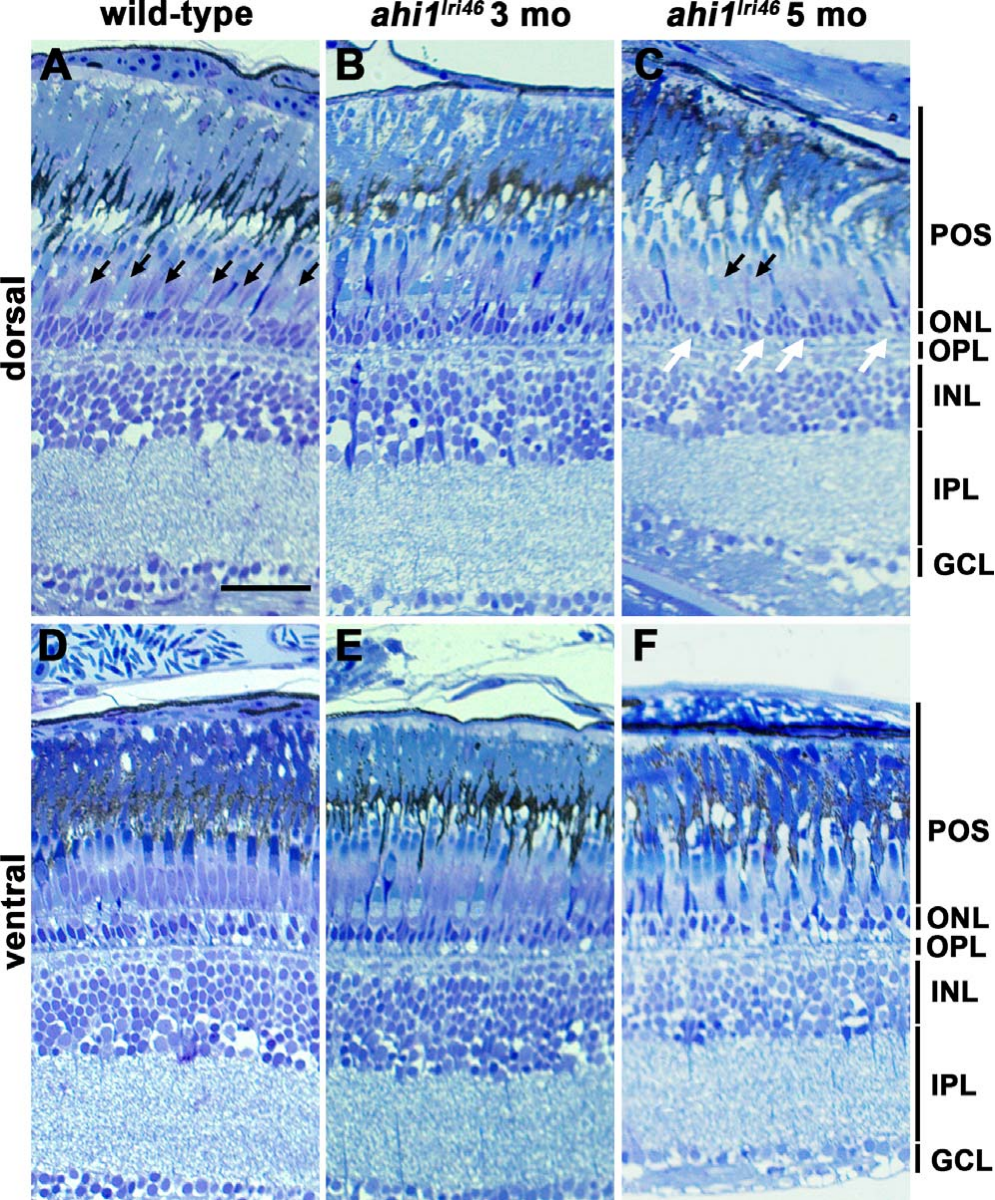
Histologic survey of retinas from wild-type and *ahi1^lri46^* adults. (**A**–**C**) Dorsal and (**D**–**F**) ventral views of toluidine blue–stained 1-μm thick sections of wild-type, 3- and 5-month-old *ahi1^lri46^* mutants. In the dorsal retina, fewer outer segments of UV-sensitive cones (*black arrows*) and thinning in the ONL (*white arrows*) was observed at 5 months of age. POS, photoreceptor outer segments; OPL, outer plexiform layer; INL, inner nuclear layer; IPL, inner plexiform layer; GCL, ganglion cell layer. *Scale bar*: 50 μm.

Roughly 2.5% of *ahi1^lri46^* mutants survived to at least 3 months of age and less than 1% survived to 5 months of age. No qualitative difference was observed in rod photoreceptor outer segments in either 3- or 5-month-old animals when compared to wild-type siblings (Figs. 4A–C). In contrast, fewer cone outer segments were observed in 5-month-old mutants (Figs. 4A, 4C, black arrows). The outer nuclear layer (ONL) in the dorsal retina of adult zebrafish is typically 3 to 4 nuclei thick (Fig. 4A). In 3-month-old *ahi1^lri46^* mutants, the ONL was largely preserved, but by 5 months it was nonuniform, with considerable thinning in some areas (Figs. 4B–C, white arrows). Interestingly, the photoreceptor phenotypes appeared confined to the dorsal retina. Images of photoreceptors in the ventral retina showed largely preserved rod and cone outer segments and a preserved ONL (Figs. 4D–F).

### Ultrastructural Analysis of Mutant Photoreceptors Reveals Abnormal Outer Segments

**Figure 5:**
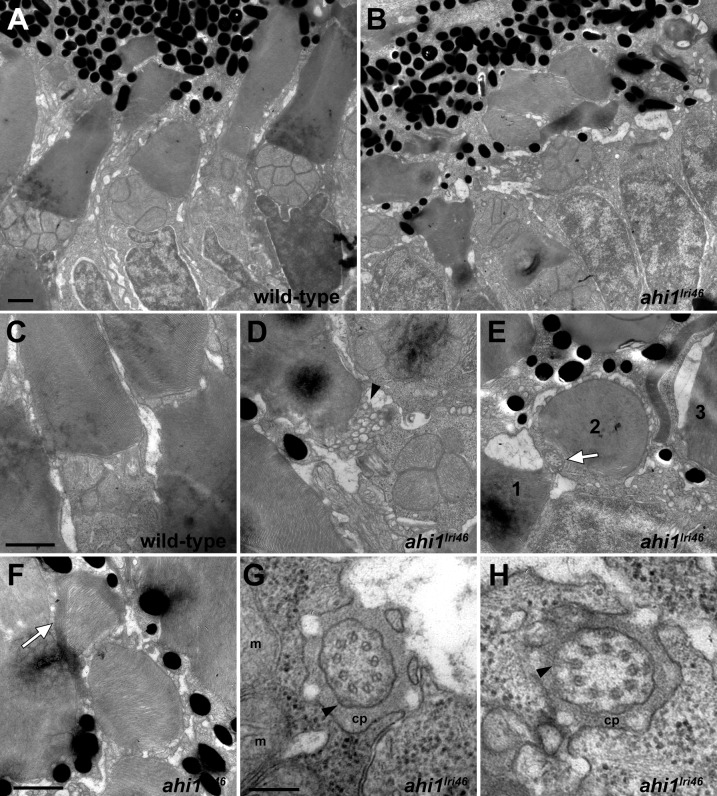
Ultrastructural analysis of wild-type and *ahi1^lri46^* photoreceptors. (**A**, **B**) Transmission electron microscopy provided ultrastructural views of wild-type and *ahi1^lri46^* photoreceptors. Mutant outer segments are short, fragmented, and disorganized. (**C**) Higher magnification of wild-type photoreceptor showing tightly stacked disc membranes and inner segment. (**D**) Vesiculation at the proximal end of an outer segment in *ahi1^lri46^* mutant (*arrowhead*). (**E**) Improper orientation of disc membranes. In this field, an axoneme (*white arrow*) and disc membranes from outer segments of three different cells, labeled 1 to 3, are oriented perpendicular to each other. (**F**) Several layers of discs are abnormally wrapped around the outer segment (*white arrow*). (**G**, **H**) Cross-sections through the ciliary pocket (cp) show the proper 9 + 0 microtubule arrangement of axoneme. Mitochondria (m) are visible in (**G**). Y-shaped crosslinkers also are present (*arrows* in [**G**, **H**]). Staining artifacts are visible as diffuse, *black deposits* in some panels. *Scale bars*: 1 μm (**A**–**F**) and 200 nm (**G**, **H**).

Disorganization of the photoreceptor outer segments was more obvious by transmission electron microscopy of 5 dpf larvae. In wild-type samples, cone photoreceptor outer segments extended toward the RPE in a parallel and organized manner, giving a palisade pattern (Figs. 5A, 5C). In contrast, the cone outer segments in *ahi1^lri46^* mutants were shorter and highly disorganized (Fig. 5B). In several instances, large amounts of vesiculated material were observed at the base of the outer segments (Fig. 5D). Occasionally, outer segments were aligned perpendicular to the plane of section, such that a transverse section produced a cross-section through the axoneme and outer segment (Fig. 5E), and in other cases we found disc membranes wrapped around the periphery of an outer segment (Fig. 5F). Such abnormalities were never observed in sections of wild-type retinas. Despite these abnormalities, we observed that the photoreceptor axonemal architecture adjacent to the transition zone was unaltered in *ahi1^lri46^* mutants when compared to wild-type controls (Figs. 5G, 5H). These results indicated that proper organization of disc membranes and outer segment morphogenesis requires Ahi1 function, but that Ahi1 is dispensable for photoreceptor ciliogenesis in zebrafish.

### Progressive Photoreceptor Degeneration in *ahi1^lri46^* Mutants

**Figure 6:**
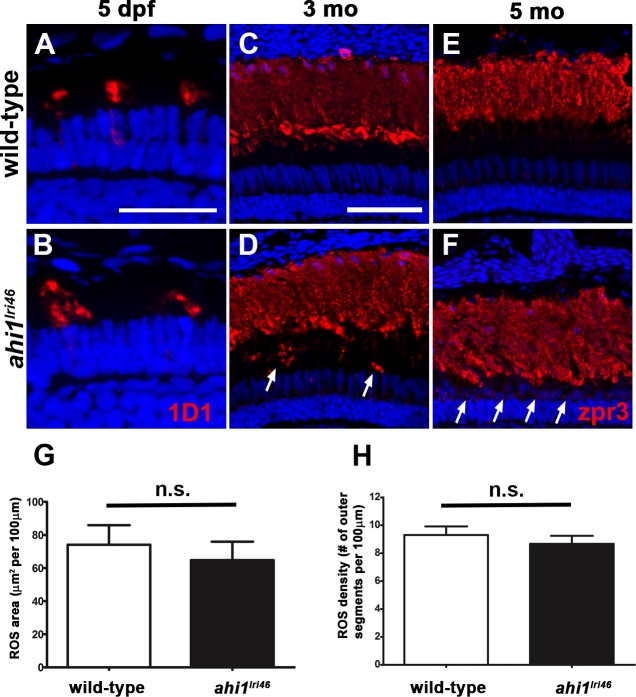
Immunohistochemical analysis of rod photoreceptors in *ahi1^lri46^* mutants. (**A**, **B**) Rod photoreceptors stained with 1D1 (*red*, rhodopsin) at 5 dpf. (**C**–**F**) Rod photoreceptors stained with zpr3 (*red*, rhodopsin) at 3- (**C**, **D**) and 5- (**E**, **F**) month-old animals. *Arrows* in (**D**) and (**F**) highlight rhodopsin mislocalization. (**G**) Quantification of rod outer segment area at 5 dpf. (**H**) Quantification of rod density at 5 dpf. *Scale bars*: 20 μm (**A**–**B**) and 50 μm (**C**–**F**).

Rod outer segment assembly requires rhodopsin transport and defects in rhodopsin trafficking have been reported for *Ahi1*-null mutant mice.^15,18^ Therefore, we hypothesized that the disorganized photoreceptors in *ahi1^lri46^* mutants would be associated with mislocalized rhodopsin. Using the monoclonal antibody 1D1, we observed that rhodopsin properly localized to the outer segments in *ahi1^lri46^* mutants at 5 dpf, comparable to wild-type siblings (Figs. 6A, 6B). Rod photoreceptor density and rod outer segment area, as determined by 1D1 staining, also were similar between wild type and *ahi1^lri46^* mutant animals (Figs. 6G, 6H). Rhodopsin mislocalization was observed in 3-month-old adults and some rhodopsin immunoreactivity was seen within the thinner ONL at 5 months (Figs. 6D, 6F, arrows). The gap between the rod outer segments and the ONL, which typically is occupied by cones, is practically absent in 5-month-old animals (Figs. 6E, 6F). No significant thinning was observed, however, in mutant rod outer segments when compared to wild-type siblings, confirming our previous observations by light microscopy.

**Figure 7:**
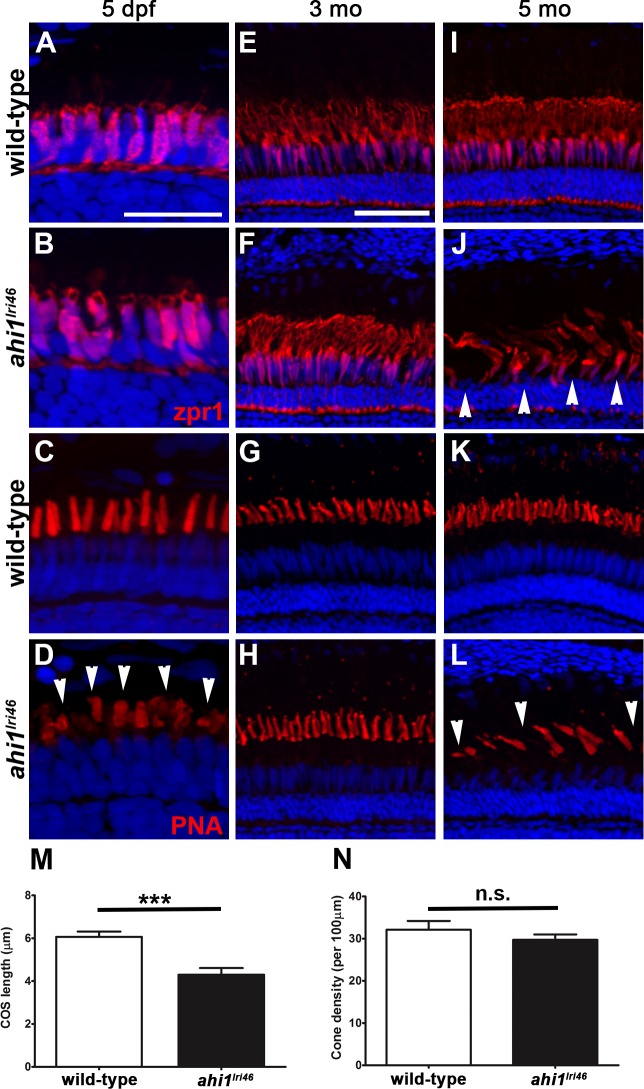
Immunohistochemical analysis of cone photoreceptors in *ahi1^lri46^* mutants. (**A**, **B**, **E**, **F**, **I**, **J**) Red-green double cones are labeled with zpr1 (*red*). Cone inner segments are preserved at 5 dpf and 3 months of age, but missing (*arrowheads*) at 5 months. (**C**, **D**, **G**, **H**, **K**, **L**) PNA staining (*red*) revealed that mutants had shorter cone outer segments ([**D**], *arrowheads*) at 5 dpf and missing at 5 months ([**L**], *arrowheads*). (**M**) Quantification of cone outer segment lengths between wild-type and *ahi1^lri46^* mutants at 5 dpf. ***P < 0.001. N, quantification of cone density at 5 dpf.

We next examined cone inner segment morphology by immunolabeling with zpr1, a monoclonal antibody that recognizes arrestin-3 on the cell bodies and inner segments of red-green double cones^31^ and with PNA to label the interphotoreceptor matrix surrounding cone outer segments.^32–34^ By zpr1 staining, the *ahi1^lri46^* cone inner segments showed a tightly packed columnar morphology, equivalent to control samples at 5 dpf (Figs.7A, 7B). Peanut agglutinin lectin staining revealed that the mutant cone outer segments were short (4.3 vs. 6.1 μm; *P* < 0.001) and misshapen at 5 dpf, consistent with our previous observation by light and electron microscopy (Figs. 7C, 7D, 7M). The total number of cone outer segments, however, was preserved (Fig. 7N), which suggested that loss of *ahi1* did not result in cell death at 5 dpf. In *ahi1^lri46^* adults, cone organization and inner segment morphology appeared normal at 3 months of age, suggesting that cones recovered in surviving animals (Figs. 7E, 7F). By 5 months of age, however, the cone inner segments were highly disorganized and appeared missing in some areas (Figs. 7I, 7J). Similarly, fewer cone outer segments were observed and the morphology of remaining outer segments was abnormal in *ahi1^lri46^* mutants at 5 months (Figs. 7G, 7H, 7K, 7L). Taken together, these data suggested that Ahi1 is required for early cone morphogenesis, yet cones survive and do not begin degenerating until at least 3 months of age.

### Visual Performance in Mutant Animals Is Not Compromised

**Figure 8:**
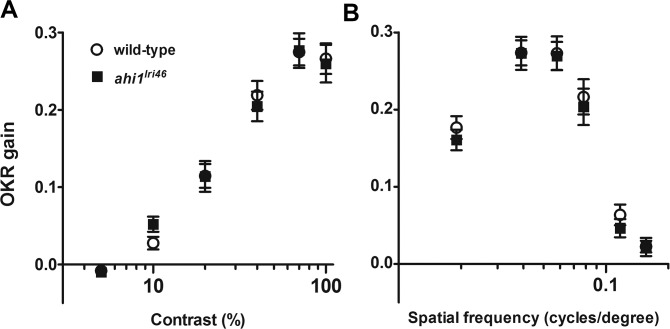
Visual function is not affected in *ahi1^lri46^* mutants at 5 dpf. Optokinetic response of wild-type (*open circles*) versus *ahi1^lri46^* homozygous mutants (*black squares*) at 5 dpf as a function of contrast (**A**) and spatial frequency (**B**) measured from smooth pursuit eye movements. Contrast sensitivity test *n* = 21 per genotype. Spatial frequency test *n* = 14 per genotype. *Error bars*: SEM.

As zebrafish visual function is cone-driven at 5 dpf^35^ and because cone outer segments were disrupted in *ahi1^lri46^* mutant retinas, we hypothesized that visual performance of *ahi1^lri46^* mutants would be compromised. We evaluated visual function by assessing the OKR gain. The OKR gain is defined as the ratio between stimulus velocity and eye velocity, and is dependent on angular stimulus velocity, spatial frequency, and contrast of the moving image.^24^ Reduced gain is indicative of defective visual performance. Using the VisioTracker system,^36^ we measured OKR gain while varying either contrast or spatial frequency.^24^ Contrast sensitivity is a general test of visual function, while spatial frequency assays under normal luminance test the ability of cones to discriminate between two stimuli (e.g., bars) separated at decreasing distances. As previously noted,^23,24^ wild-type animals show a linear relationship between gain and the logarithm of contrast. Optokinetic response gain in mutant animals was not significantly different from wild-type controls in response to varying either the stimulus contrast or spatial frequency (Fig. 8), and, therefore, we concluded that visual function in these animals is preserved.

### Loss of Ahi1 Does Not Affect Components of the Connecting Cilium

**Figure 9:**
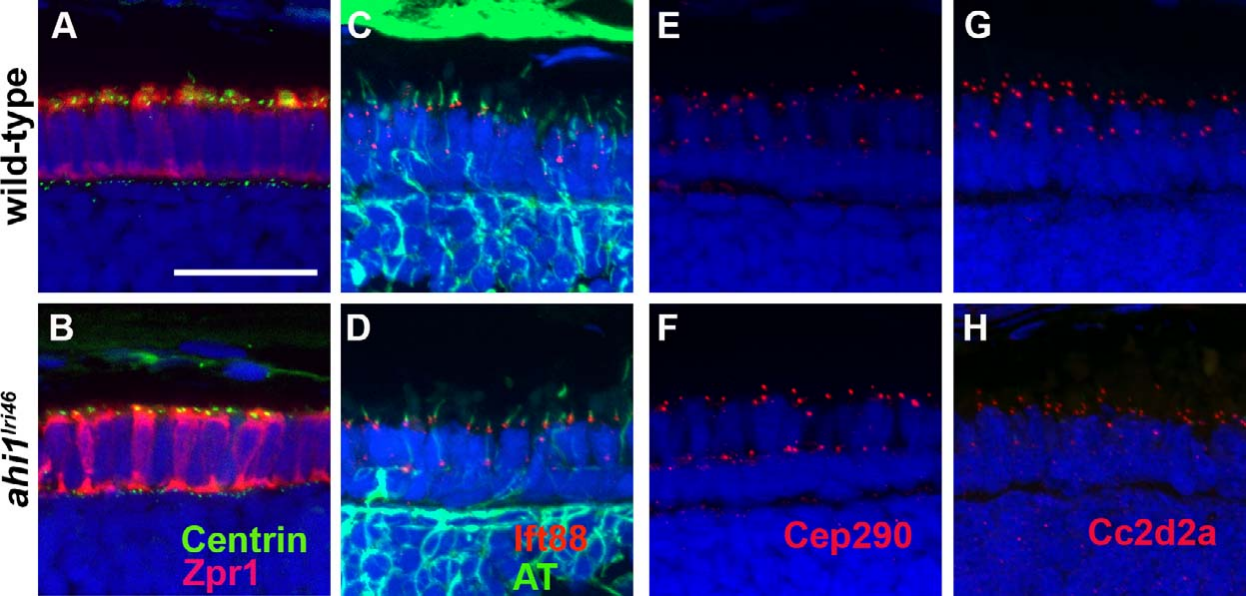
*ahi1* is not required for ciliogenesis or transition zone protein localization. (**A**, **B**) *Tg(centrin:GFP)* expression (*green*) localized to the apical inner segments in wild-type and *ahi1^lri46^* mutants at 3 dpf. Zpr1 (*red*) was used to stain cone photoreceptors to visualize the apical boundary. (**C**, **D**) Five dpf retinas stained with acetylated tubulin (*green*) and Ift88 (*red*) showed ciliary localization of the IFT particle in wild-type and mutant animals. (**E**, **H**) The transition zone proteins Cep290 and Cc2d2a (*red*) exhibited punctate staining in wild-type and mutant animals. *Scale bar*: 20 μm.

As Ahi1 localizes to the basal body in cultured cells,^17^ we next interrogated whether loss of Ahi1 affected the localization of components of the basal body and axoneme. We first examined basal body localization using the *Tg(centrin-GFP)* transgenic line, which expresses a centrin-GFP fusion protein.^20^ Centrin is a calcium-binding protein that localizes to basal bodies.^37^ Similar to observations in *Ahi1-*null mice,^18^ basal bodies localized normally to the apical pole of the inner segments of wild-type and *ahi1^lri46^* mutant photoreceptors (Figs. 9A, 9B). We next evaluated the expression of Ift88, a component of the IFT machinery, and acetylated tubulin, to determine if IFT particles localized to the connecting cilium. In wild-type and *ahi1^lri46^* mutant photoreceptors, Ift88 immunoreactivity was observed as punctate foci at the base of the connecting cilium (Figs. 9C, 9D).

The transition zone (TZ) refers to the region at the proximal end of the cilium and functions as a ciliary gate to regulate protein entry. The Cep290 and Cc2d2a proteins colocalize to the transition zone and are two components of a larger MKS complex.^38–40^ Mutations in those genes also lead to JBTS in humans.^41,42^ Using a rabbit polyclonal antibody against the N-terminus of Cep290, we detected punctate immunoreactivity in the region consistent with the connecting cilium of wild-type and *ahi1^lri46^* mutants (Figs. 9E, 9F). When immunostaining for Cc2d2a was performed, we observed strong punctate labeling in the connecting cilium of wild-type samples (Fig. 9G). In *ahi1^lri46^* mutants, Cc2d2a immunoreactivity was less intense than what was observed for wild-type samples, but the localization was normal (Fig. 9H). These results indicated that the absence of Ahi1 does not compromise the localization of these proteins to the transition zone, but may reduce Cc2d2a stability at the transition zone.

### Zebrafish *ahi1* Is a Functional Ortholog of Human *AHI1*

**Figure 10:**
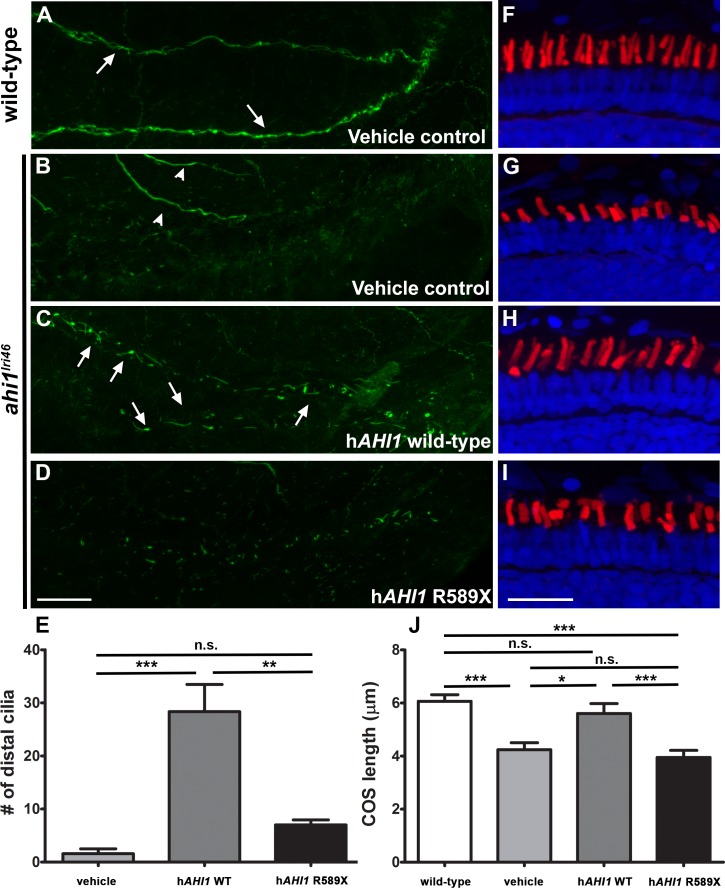
Human *AHI1* RNA rescues *ahi1^lri46^* mutant phenotype. (**A**–**D**) Whole-mount immunolabeling of pronephric cilia with anti-acetylated tubulin (*green*) at 36 hpf. Cilia are seen in pronephric ducts of wild-type animals and in *ahi1^lri46^* mutants following injection of mRNA encoding human *AHI1* ([**A**, **C**]; arrows). Motor neurons also stained with acetylated tubulin ([**B**], arrowheads). (**E**) Quantification of cilia numbers in the distal pronephric duct of *ahi1^lri46^* mutants following injection. Distal pronephric cilia in wild-type animals were too numerous to count and are not presented graphically. (**F**–**I**) Images of 5 dpf retinas stained with PNA (*red*) and DAPI (*blue*) to label cone outer segments. (**J**) Quantification of cone outer segment lengths (*n* = 6). *Scale bar*: 20 μm.

To confirm that the observed phenotypes were due to loss of *ahi1*, as well as to determine whether the *ahi1^lri46^* mutant could be used to functionally test human disease alleles, wild-type and *ahi1^lri46^* embryos were injected with 100 pg of mRNA corresponding to either the human wild-type *AHI1* or the *AHI1^r589X^* mutant. The R589X mutation is associated with retinal degeneration in Joubert patients^11,13^ and occurs upstream of the first WD40 repeat. This mutation is predicted to eliminate all WD40 repeats and the SH3 domain of the protein, which is similar to the predicted effects of the truncating mutation in the *ahi1^lri46^* mutant. Overexpression of wild-type *AHI1* did not cause any morphologic abnormalities in wild-type embryos (data not shown). Injection of wild-type human *AHI1* mRNA into the *ahi1^lri46^* mutants, however, significantly rescued the mutant phenotypes. Injection of 100 pg of wild-type *AHI1* mRNA resulted in increased numbers of cilia in the distal kidney of *ahi1^lri46^* mutants when compared to embryos injected with vehicle or those injected with the *AHI1^r589X^* mRNA (Figs. 10A–E). Similarly, the cone outer segments of 5 dpf *ahi1^lri46^* mutants were of comparable length to wild-type siblings following injection of wild-type *AHI1* mRNA while cone outer segments in mutant fish injected with vehicle or the *AHI1^r589X^* mRNA remained shorter (Figs. 10F–J; *n* = 6 animals per condition). Together, these data supported the contention that *ahi1* is a functional ortholog of human *AHI1*, and that the phenotype observed in our mutant line is due to *ahi1* deficiency.

## Discussion

Joubert syndrome is a ciliopathy that has been associated with mutations in approximately 27 cilia genes and has a wide range of clinical presentations.^8,11,13^ *AHI1* was the first gene associated with Joubert syndrome,^12,14^ and, together with *CEP290*, it is one of the genes most commonly associated with retinal dystrophy and kidney disease in JBTS patients.^8,11^ Here, we report the generation of *ahi1^lri46^* mutant zebrafish that exhibit retinal dystrophy and loss of distal pronephric duct cilia. This mutant could function as a new model to study the ocular and renal dysfunction present in patients with JBTS.

Studies on cultured cells have shown that Ahi1 associates with the basal body,^17^ and localization experiments performed in a mouse model further localized Ahi1 to the transition zone of the photoreceptor sensory cilium.^18^ Furthermore, primary cilia were absent in Ahi1-knockdown cells as well as in *Ahi1^–/–^* mouse embryonic fibroblasts, supporting the idea that Ahi1 may have an important role in primary cilia formation and maintenance.^15,17,18^ One of the proposed mechanisms in how Ahi1 might function during ciliogenesis is through its interaction at the basal body with Rab8a, a GTPase that has been identified as a critical modulator of ciliogenesis.^43,44^ In this report we showed in zebrafish that loss of *ahi1* does not completely abolish ciliogenesis, but rather affects photoreceptor outer segment morphogenesis and maintenance. The significant accumulation of disorganized vesiculated material observed at the basal domain of photoreceptor outer segments, in what seems to be an attempt to form disc membranes, opens the possibility that *ahi1* has a supporting role in disc morphogenesis.

Mice with a targeted deletion of *Ahi1* had high mortality at birth.^15,18^ Among the surviving animals, photoreceptors lacked outer segments by postnatal day 12, but they retained axonemes and connecting cilia.^15,18^ The zebrafish *ahi1^lri46^* mutant, although ultimately lethal, survives until 10 dpf, and has a significantly milder retinal phenotype, in that outer segments are produced and the animals have a robust OKR. This discrepancy between the two models may reflect the presence of maternally derived *ahi1* RNA in developing larvae, which we detected in zygote-stage embryos by in situ hybridization. The lack of a suitable antibody recognizing zebrafish Ahi1 precludes a direct test of protein levels in the mutant. One significant parallel between the two models, however, is the presence of disorganized vesiculated material at the distal end of the transition zone, which supports the contention of a role for Ahi1 in photoreceptor disc morphogenesis. Although findings from cell culture experiments suggested a role for *Ahi1* in ciliogenesis,^17^ the mouse mutant and our zebrafish mutant exhibit abnormal cilium-associated structures (e.g., photoreceptor outer segments), while the microtubule arrangement within the axoneme appears normal. This suggests to us that *Ahi1* is dispensable for rudimentary photoreceptor ciliogenesis. Our observation that ciliary markers, such as Centrin, Ift88, Cep290, and Cc2d2a, are properly localized further supports this conclusion.

One notable difference between the mouse and zebrafish models is that, unlike the mouse, zebrafish rod outer segments are preserved. In humans with ciliopathies as well as in mouse models of those diseases, rod degeneration often occurs first, invariably followed by a secondary degeneration of cones.^45,46^ Yet, despite the compromised cone outer segment morphology at 5 dpf, the mutant's robust optokinetic response (which is completely cone-derived at this age) indicates that these abnormal cells remain functional.

In contrast to what was observed in photoreceptors, we show that loss of *ahi1* leads to the loss of pronephric duct cilia, implying that the role of *ahi1* may vary among tissues, and that it may be more critical in the kidney. We speculate that Ahi1 is a component of a larger complex in the basal body, and that the precise composition of this complex is tissue-dependent. Therefore, loss of Ahi1 in one tissue type may be more destabilizing to the complex as a whole in certain tissues. Furthermore, it has been shown that multiciliated cells are more prominent at the proximal end of the pronephric duct, whereas monociliated cells predominate at the distal end.^47^ This uneven distribution of cells could be behind the uneven depletion of pronephric duct cilia in *ahi1^lri46^* mutant zebrafish.

We demonstrated that zebrafish *ahi1* is a functional ortholog of human *AHI1* and that the *ahi1^lri46^* allele could be used to functionally test human disease alleles. Partial and complete rescue of the renal and retinal phenotypes, respectively, was observed after the injection of wild-type human *AHI1* RNA. Such rescue was not observed after the injection of mutant human *AHI1* RNA, confirming that human disease alleles of *AHI1* can be easily studied in zebrafish.

In summary, to our knowledge this is the first report to present an *ahi1* mutant zebrafish and suggested an important role of *ahi1* in photoreceptor outer segment formation and maintenance, as well as in morphogenesis of distal pronephric duct cilia, making it a possible in vivo model for the oculo-renal component of Joubert syndrome.

## Supplementary Material

Supplement 1
