# Supplementary material for: The Ciliopathy Gene ahi1 Is Required for Zebrafish Cone Photoreceptor Outer Segment Morphogenesis and Survival
Source: Invest Ophthalmol Vis Sci. 2017 Jan;58(1):448–60. doi: 10.1167/iovs.16-20326 (PMC5270624; doi:10.1167/iovs.16-20326)
Supplement: Supplement 1 [file iovs-58-01-18_s01.pdf]

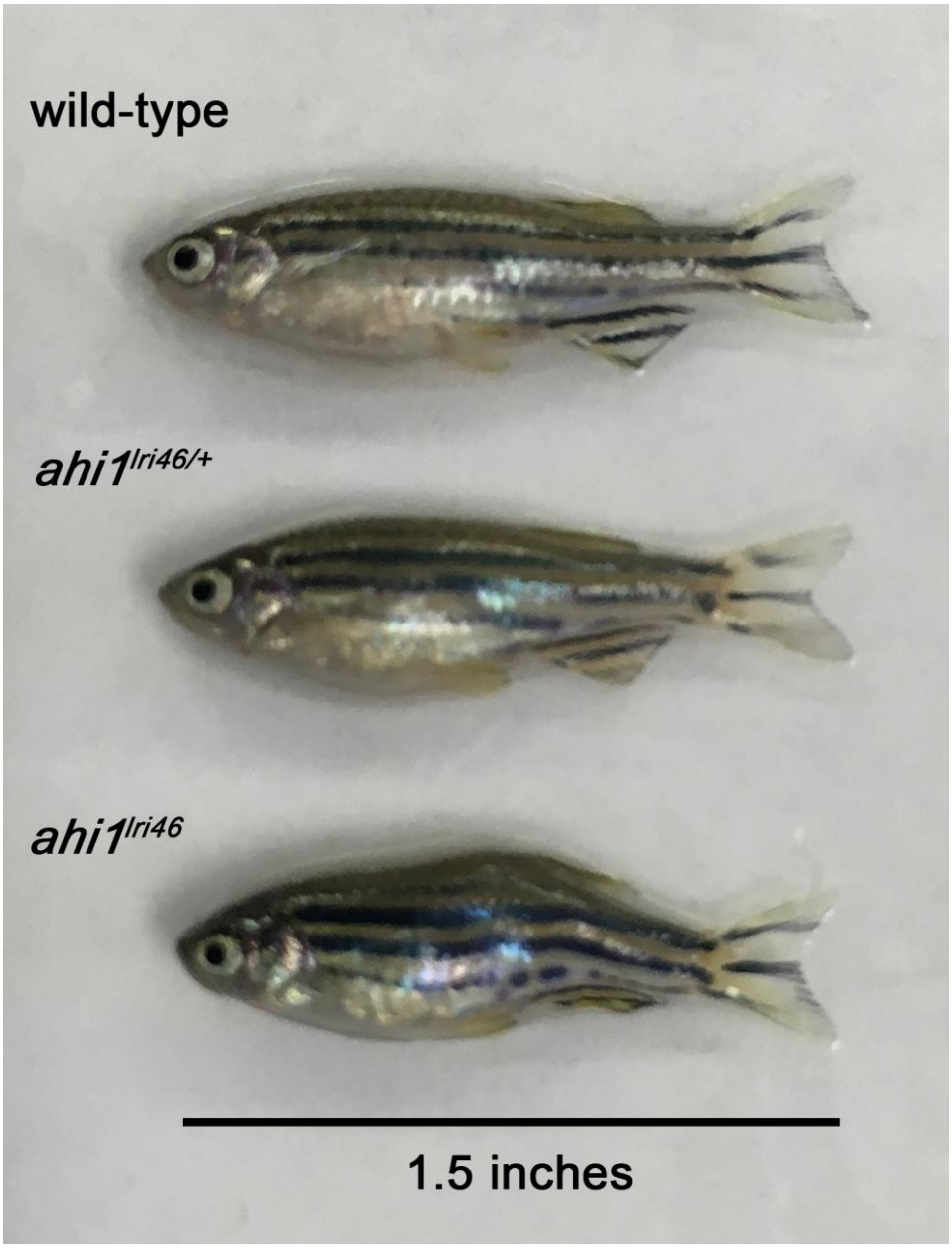

Supplemental Figure 1. Zebrafish *ahi1* mutants have a characteristic phenotype.

Lateral view of 5-month-old wild-type, heterozygous *(ahi1$^{lri46/+}$)* and homozygous *ahi1^{lri46}* mutant displaying scoliosis of the vertebral column.
